# Does Male Circumcision Reduce Women's Risk of Sexually Transmitted Infections, Cervical Cancer, and Associated Conditions?

**DOI:** 10.3389/fpubh.2019.00004

**Published:** 2019-01-31

**Authors:** Brian J. Morris, Catherine A. Hankins, Joya Banerjee, Eugenie R. Lumbers, Adrian Mindel, Jeffrey D. Klausner, John N. Krieger

**Affiliations:** ^1^School of Medical Sciences and Bosch Institute, University of Sydney, Sydney, NSW, Australia; ^2^Faculty of Medicine, McGill University, Montreal, QC, Canada; ^3^London School of Hygiene and Tropical Medicine, Bloomsbury, London, United Kingdom; ^4^Jhpiego, Washington, DC, United States; ^5^School of Biomedical Sciences and Pharmacy, Faculty of Health and Medicine and Priority Research Centre for Reproductive Science, University of Newcastle, Callaghan, NSW, Australia; ^6^Mothers and Babies Research Centre, Hunter Medical Research Institute, New Lambton, NSW, Australia; ^7^Sydney Medical School, University of Sydney, Sydney, NSW, Australia; ^8^Division of Infectious Diseases and the Program in Global Health, Fielding School of Public Health, University of California Los Angeles Care Center, Los Angeles, CA, United States; ^9^Department of Urology, University of Washington School of Medicine, Seattle, WA, United States

**Keywords:** women, circumcision, male, sexually transmitted infections, HIV, HPV, public health, preventive medicine

## Abstract

**Background:** Male circumcision (MC) is proven to substantially reduce men's risk of a number of sexually transmitted infections (STIs). We conducted a detailed systematic review of the scientific literature to determine the relationship between MC and risk of STIs and associated conditions in women.

**Methods:** Database searches by “circumcision women” and “circumcision female” identified 68 relevant articles for inclusion. Examination of bibliographies of these yielded 14 further publications. Each was rated for quality using a conventional rating system.

**Results:** Evaluation of the data from the studies retrieved showed that MC is associated with a reduced risk in women of being infected by oncogenic human papillomavirus (HPV) genotypes and of contracting cervical cancer. Data from randomized controlled trials and other studies has confirmed that partner MC reduces women's risk not only of oncogenic HPV, but as well *Trichomonas vaginalis*, bacterial vaginosis and possibly genital ulcer disease. For herpes simplex virus type 2, *Chlamydia trachomatis, Treponema pallidum*, human immunodeficiency virus and candidiasis, the evidence is mixed. Male partner MC did not reduce risk of gonorrhea, *Mycoplasma genitalium*, dysuria or vaginal discharge in women.

**Conclusion:** MC reduces risk of oncogenic HPV genotypes, cervical cancer, *T. vaginalis*, bacterial vaginosis and possibly genital ulcer disease in women. The reduction in risk of these STIs and cervical cancer adds to the data supporting global efforts to deploy MC as a health-promoting and life-saving public health measure and supplements other STI prevention strategies.

## Introduction

Sexually transmitted infections (STIs) are an increasing threat to global health. The World Health Organization (WHO) found, “*more than 1 million* [STIs] *are acquired every day worldwide. Each year, there are an estimated 357 million new infections with* [….] *chlamydia, gonorrhoea, syphilis and trichomoniasis. More than 500 million people are estimated to have genital infection with herpes simplex virus (HSV). More than 290 million women have a human papillomavirus (HPV) infection*” (1). The WHO emphasizes that STIs in women often have no symptoms, cause adverse birth outcomes, can increase susceptibility to HIV infection, can have long-term morbidity, exhibit drug resistance in the case of gonorrhea, and can cause infertility, risk mother-to-child transmission of STIs, and increase premature mortality (1). HIV infection is associated with increased risk of a wide variety of co-morbidities (2). Prevalence of STIs in the US is high (3). On Aug 28, 2018, the CDC estimated a further 200,000 annual STI cases in the US in 2017, warning of a “*public health crisis*” (4).

For HPV, global prevalence is 11.7%, highest prevalence being in sub-Saharan Africa (24%), Eastern Europe (21%), and Latin America (16%) (5). There are ~200 HPV genotypes, including over 40 that occur in the anogenital region (6). The 2013–2014 National Health and Nutrition Examination Survey (NHANES) found a 44.8% prevalence of one or more of 37 HPV genotypes in 2,174 women aged 18–39 years (7).

Virtually all cases of squamous cell carcinoma of the cervix are caused by oncogenic (“high-risk”) HPV genotypes (8–11). Cervical dysplasia is the first visible abnormality, and is a precursor to cervical cancer. Most often it will resolve, but slow progression to cervical cancer may occur. While screening by pap smears (and recently by direct HPV testing) has, over the decades, reduced cervical cancer in wealthy countries, cervical cancer continues to be a major cause of death in women in low income countries (12). New biomarkers (12), such as p16^INK4a^ (13), a methylation signature (14), and nodal metastasis detection techniques (15) have shown promise for screening triage. This includes in elderly women (16). Co-factors for dysplasia can include genetics or lifestyle, especially smoking. In 2015, there were 526,000 diagnoses and 239,000 deaths from cervical cancer worldwide (17). Ninety percent of these deaths occur in low- and middle-income countries due to the scarcity of HPV screening and affordable treatment (18). In the US, 13,240 new diagnoses and 4,170 deaths from cervical cancer were predicted for 2018 (19). Cervical cancer caused 7 million disability adjusted life years (DALYs, i.e., the number of years of life lost due to ill-health, disability or early death) in 2015, with 96% coming from years of life lost and 4% from years lived with disability (17). For all cancers in low-income countries, the relevant DALYs lost to cervical cancer are second only to oropharyngeal cancer (2,252) (20).

Besides the cervix, HPV-associated invasive cancers in women occur in the vulva, vagina, oropharynx, anus and rectum (21). In the US, there are 35,000 HPV-associated cancers annually (61% in females and 39% in males), with an annual cost for screening and treatment of HPV-associated health outcomes of US$8 billion (22). Beyond the direct social costs, costs to the health system, physiological impact, and psychological impacts on individuals and families are the indirect costs, such as loss of current and future earnings and cost to others for child care provision.

For HSV-2, global prevalence of people aged 15–49 years living with this infection exceeds 417 million, with incident infections being 12 million in women and 7 million in men in 2012 (23). HSV-2 prevalence in the US, according to 2013–2014 US NHANES data amongst 2,174 women aged 18–39 years was 14.3% (39.8% in African-American women and 10.5% in women of other races/ethnicities) (7).

*Chlamydia trachomatis* is the second most frequent STI globally after HPV and is the most common bacterial STI. The WHO estimates that there are 92 million new cases annually, of which 3-million occur in the USA, at an annual cost for care of $2 billion (24).

The 2013–2014 US NHANES data for urinary *C. trachomatis* prevalence among 2,174 women aged 18–39 years was 2.2% (5.4% among African-American women and 1.7% among women of other races/ethnicities) (7). In 2017 there were 687 cases per 100,000 US females of all ages, with rates rising 6.5 and 3.7% among females aged 15–19 and 20–24 years, respectively (3). Prevalence has increased each year since 2000, being twice as high in females as in males (25). Untreated *C. trachomatis* in women causes pelvic inflammatory disease in up to 10% of women, with inflammatory damage to fallopian tubes accounting for 30% of female infertility in the US, and can cause ectopic pregnancy (3). *C. trachomatis* infection represents a co-factor for HPV-induced cervical cancer (26) and, in both sexes, HIV transmission. A recent serology study in two independent populations found antibodies against *C. trachomatis* (Pgp3) to be associated with increased ovarian cancer risk (27).

Gonorrhea cases have also risen in the US, with a 33% increase in women during 2015–2017 to 142 cases per 100,000 females, peaking at 872 cases per 100,000 among 19-year olds (3). Over time, gonorrhea resistance to common antimicrobials used to treat it has also increased, leading the WHO to refer to gonorrhea as a “superbug” untreatable by common once-effective antibiotics, such as ciprofloxacin, that were once effective treatments (28).

*T. vaginalis* is another common STI worldwide. The US 2013–2014 NHANES data for 4,057 females aged 18–59 found a *T. vaginalis* prevalence of 1.8% (prevalence being 8.9% among African-American women, a population at high risk of STIs, and 0.8% among other ethnicities) (7). Prevalence was 3.6-fold greater for women with ≥2 vs. 0–1 sexual partners in the past year (7). *T. vaginalis* prevalence among women in suburban Sydney, Australia, was 3.4% (29). *T. vaginalis* infection upregulates inflammation in synergy with *Mycoplasma hominis*, which could increase risk of cervical cancer and HIV acquisition (30).

Syphilis prevalence in women globally is 0.56%, with data from 132 countries revealing that more countries faced increases rather than reductions between 2012 and 2016 (31). A 2018 WHO report estimated that, “*over 900,000 pregnant women were infected with syphilis resulting in approximately 350,000 adverse birth outcomes including stillbirth in 2012*” (1). During 2013–2017, primary and secondary syphilis rate in US women increased by 156% from 0.9 to 2.3 cases per 100,000 females (3), risking fatalities, stillbirths in pregnant women, prematurity and congenital infections (32). In the US, syphilis is responsible for 4.2 million DALYs (32).

Of total HIV diagnoses in women in 2016 in the US, heterosexual acquisition accounted for 63% (6,541 cases) (33). By 2016, 154,584 US women had stage 3 HIV infection (AIDS) (33). In Australia, HIV infections among women have risen steadily, with heterosexual intercourse responsible for 25% of HIV diagnoses in 2017 (34). Sixty six percent of these involved sexual intercourse with a person who was not in or from a high-prevalence country. A strong positive correlation between HIV prevalence and wealth has been documented in Kenya, where HIV prevalence among women is 4% in the lowest economic quartile and 12% in the highest (35). Men, who are more likely than women to have multiple sexual partners, are the likely source of infection for most women (36). Without effective antiretroviral treatment supressing HIV replication, an infected woman may pass HIV on to her offspring during pregnancy and breastfeeding.

Bacterial vaginosis (BV; formerly termed gardnerella and “non-specific vaginitis”) is one of the most common genital infections in women worldwide. BV prevalence ranges from 8 to 75% in different countries (37). In the US, prevalence ranges from 15 to 49%, being higher in women with ≥ 4 sexual partners (38). A high level of resistance to commonly-used antibiotics has been observed (38). BV symptoms may include a watery, white or gray discharge instead of normal vaginal secretions, and a strong or unusual “fishy” odor from the vagina. BV is characterized by a shift in the composition of vaginal microbial communities from the normal healthy bacteria—in particular lactic acid-producing lactobacilli—with an overgrowth of various other bacteria, notably strict anaerobes, and an elevation in pH (alkalinity) of vaginal fluid. BV is regarded by some as sexually transmitted (39), with an epidemiology similar to that of established STIs (39, 40). A meta-analysis found an association of BV with a 51% higher prevalence of cervical pre-cancerous lesions (OR 1.5l; 95% CI 1.24–1.83) (41).

Commitment to control all STIs requires dispassionate public health measures to replace prevention based simply on, “*moral prophylaxis*” (32). When used consistently and correctly, condoms offer level 2 evidence of effectiveness—i.e., evidence obtained from well-designed controlled trials without randomization and well-designed cohort or case-control analytical studies preferably by more than one center or research group (42)—of at least partial (43, 44) protection against most, but not all, STIs (32).

Scientific evidence consistently points to the vulnerability of the male prepuce (foreskin) to infection (45). Not only does the preputial cavity trap microorganisms, the preputial space has an aerobic microbiome and a large mucosal surface containing target cells for infectious agents (45). The dermal preputial surface is particularly fragile, facilitating establishment, and persistence of STIs (46).

Over recent years, there has been a considerable increase in the quantity and quality of scientific evidence documenting the safety and substantial health benefits of male circumcision (MC), especially when performed early in infancy (43, 47–50), when benefits exceeded risks by over 100 to one (44, 51). Benefits start with protection against urinary tract infections (52, 53), especially in congenital urinary system anomalies, such as vesicoureteral reflux (52). Benefits to men include reducing the risk of certain STIs (54–58). Early evidence for protection afforded by MC against heterosexually acquired HIV infection in men (59–63) has now been proven at the highest standards of scientific evidence, including 3 large well-designed randomized controlled trials (RCTs) in sub-Saharan Africa (64–66), a systematic review (67), and a Cochrane committee meta-analysis of the MC trial results (68). This led to MC being endorsed by the WHO and UNAIDS as an additional important intervention to help reduce HIV incidence in epidemic settings (69). Roll-out of voluntary medical MC (VMMC) programs has resulted in 18.9 million MC procedures in high-priority countries (70), helping reduce infections and save lives (71). The MC RCTs subsequently found risk reductions for several other STIs in men (57). As a result of MC benefits to males now being well-established, affirmative policy recommendations were developed by the US Centers for Disease Control and Prevention (CDC) (58) and by the American Academy of Pediatrics (48, 72), the latter policy being endorsed by the American College of Obstetrics and Gynecology.

A lower STI prevalence in males would be expected to lead to a lower prevalence in women, sufficient to demonstrate a positive effect on female public health (73). VMMC has been very effective in lowering HIV infections in both sexes in epidemic settings of sub-Saharan Africa (74–78), by as much as 50% in a recent Kenyan study (78), and there is a correlation between MC and population prevalence of HIV in both sexes (79), although limitations exist for such ecological observations (80). In a study of serodiscordant couples, protection afforded by MC was greatest when HIV viral load was high (81).

Here we present the results of a detailed systematic review of the scientific evidence concerning the impact of a man's circumcision status on the STI risk and its subsequent impact on the genital health of his female sexual partners.

## Methods

**Figure 1:**
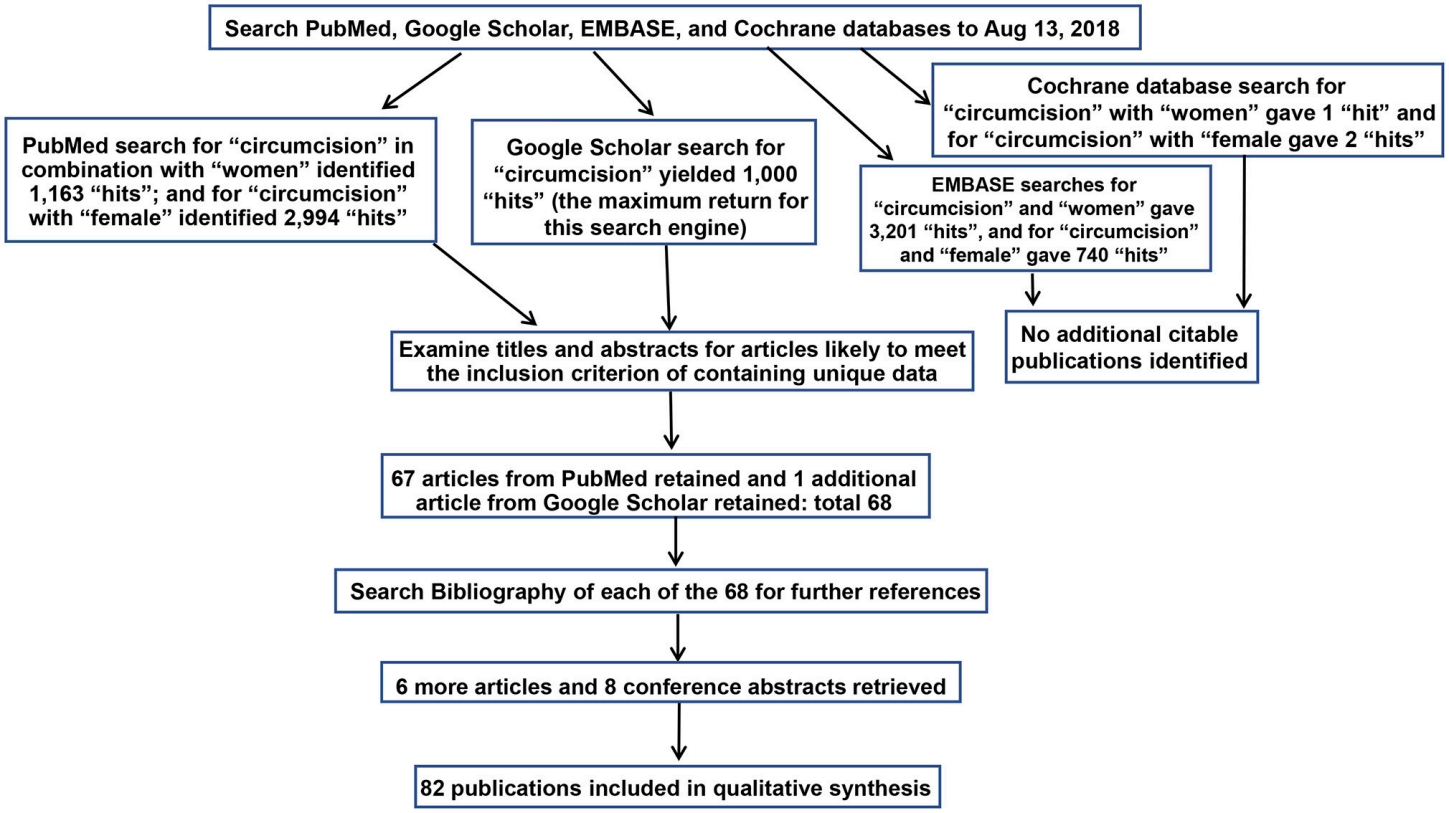
Search strategy diagram as required by PRISMA guidelines (151).

**Figure 2:**
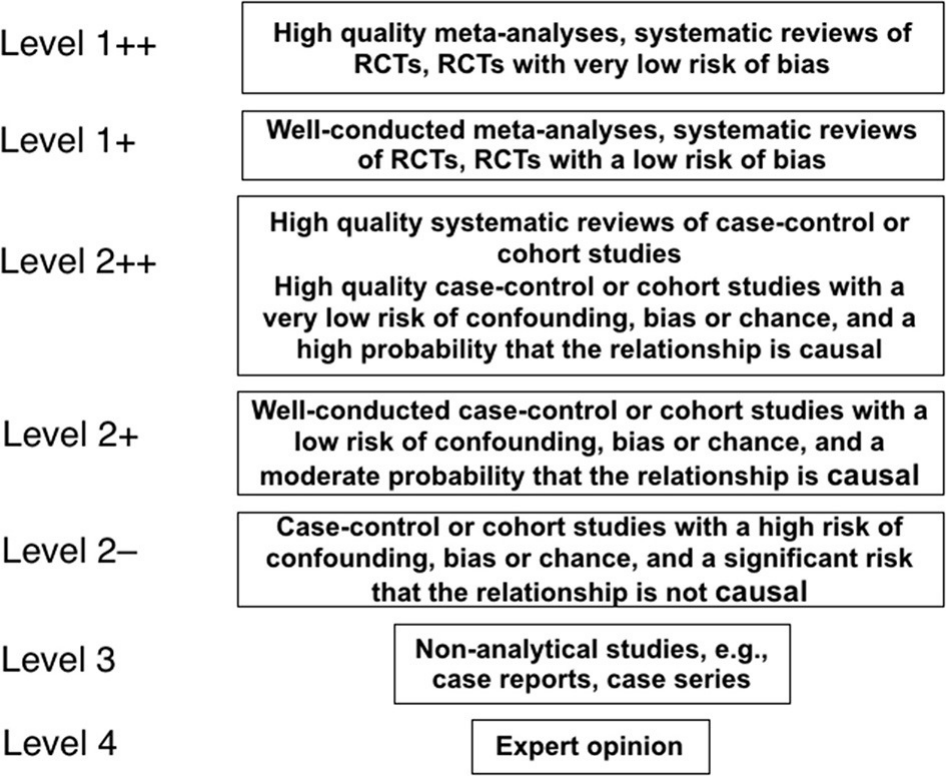
The hierarchy of quality of evidence used in science to evaluate claims, as specified by the Scottish Intercollegiate Guidelines Network (42).

Articles were retrieved through sequential searches of PubMed, Google Scholar, EMBASE and the Cochrane database of Systematic Reviews using the keywords (i) “circumcision” and “women” and (ii) “circumcision” and “female” on Aug 13, 2018 for publications pertaining to STIs and associated conditions in women. The particular STIs and conditions are listed in Table 1. Results already identified in previous searches were not included again. Titles and abstracts were examined, and the full texts of articles with the potential to meet the inclusion criteria were examined. Figure 1 shows the search strategy in accord with PRISMA guidelines (151). Articles were assessed for quality and those rated “2–” and above by the Scottish Intercollegiate Guidelines Network (SIGN) grading criteria (Figure 2) (42) were studied further. The most relevant and representative of the topic were then cited. Bibliographies were examined to retrieve further key references.

**Table 1:** Publications on effect of male circumcision on cervical cancer and STIs in female partners,^*^ together with quality rating^**^.

Rating^**^	**Condition**
**CERVICAL CANCER**	
1++	Bosch et al. (82)
2++	Castellsague et al. (83), Drain et al. (84)
2+	Boyd and Doll (85), Brinton et al. (86), Kjaer et al. (87), Aung et al. (88), Kim et al. (89)
2–	Braithwaite (90), Plaut and Kohn-Speyer (91), Pratt-Thomas et al. (92), Heins and Dennis (93), Reddy and Baruah (94), Terris et al. (95), Agarwal et al. (96), Dhar et al. (97), Gajalakshmi and Shanta (98), Svare et al. (99), Yasmeen et al. (100), Al-Awadhi et al. (101), Shavit et al. (102)
**CERVICAL DYSPLASIA**	
2+	Dajani et al. (103), Fonck et al. (36), Kim et al. (89), Soh et al. (104)
2–	Kolawole et al. (105)
**HPV INFECTION**	
1++	Wawer et al. (106), Lei et al. (107), Grabowski et al. (108), Tobian et al. (109)
2++	Roura et al. (110)
2–	Kolawole et al. (105)
**HIGH-RISK HPV INFECTION**	
1++	Tobian et al. (109), Wawer et al. (106)
2+	Obiri-Yeboah et al. (111)
**HIGH-RISK HPV VIRAL LOAD**	
1++	Grabowski et al. (108)
1+	Senkomago et al. (112), Davis et al. (113)
**LOW-RISK HPV INFECTION**	
1++	Tobian et al. (109), Wawer et al. (106)
**GENITAL WARTS**	
2+	Fonck et al. (36)
**HERPES SIMPLEX VIRUS TYPE 2**	
1++	Tobian et al. (114)
2++	Mugo et al. (115)
2+	Cherpes et al. (116), Drain et al. (84), Borkakoty et al. (117), Mujugira et al. (118), Davis et al. (119)
2–	Mehta et al. (120)
**NON-SPECIFIC GENITAL ULCERS**	
2+	Fonck et al. (36), Brankin et al. (121), Tobian et al. (122)
**GENITAL ULCER DISEASE**	
1++	Gray et al. (123)
***Chlamydia trachomatis***	
2++	Castellsague et al. (124)
2+	Fonck et al. (36), Turner et al. (125), Russell et al. (126)
2–	Nayyar et al. (127), Moodley et al. (128)
**GONORRHEA**	
2++	Turner et al. (125)
2+	Fonck et al. (36)
2–	Nayyar et al. (127), Moodley et al. (128)
***Trichomonas vaginalis***	
1++	Gray et al. (123), Wawer et al. (129)
2+	Fonck et al. (36), Turner et al. (125)
**SYPHILIS**	
1+	Pintye et al. (130)
2++	Moodley et al. (128), Davis et al. (119)
2+	Fonck et al. (36), Lawi et al. (131)
2–	Nayyar et al. (127)
**HIV INFECTION**	
1+	Wawer et al. (129)
2++	Chao et al. (132), Hunter et al. (133), Kapiga et al. (134), Turner et al. (135), Babalola (136), Baeten et al. (137), Poulin and Muula (138), Hughes et al. (139), Jean et al. (140), Auvert et al. (141), Davis et al. (119), Fatti et al. (142)
2+	Allen et al. (143), Fonck et al. (36), Mapingure et al. (144), Chemtob et al. (145), Cuadros et al. (79), Lawi et al. (131), Fox and Noncon (146)
**BACTERIAL VAGINOSIS**	
1++	Gray et al. (123), Wawer et al. (129), Tobian et al. (147)
2++	Cherpes et al. (148)
2+	Zenilman et al. (149), Fonck et al. (36)
2–	Nayyar et al. (127), Schwebke and Desmond (150)
***Mycoplasma genitalium***	
1++	Tobian et al. (122)
**CANDIDIASIS**	
2+	Fonck et al. (36)
**DYSURIA**	
2++	Gray et al. (123), Wawer et al. (129)
**VAGINAL DISCHARGE**	
2++	Gray et al. (123), Wawer et al. (129), Tobian et al. (122)

* *For women with circumcised vs. women with uncircumcised sexual partners*.

** *Quality rating was based on the SIGN international grading system (42). 1++ High quality meta-analyses, systematic reviews of RCTs, or RCTs with a very low risk of bias; 1+ Well conducted meta-analyses, systematic reviews of RCTs, or RCTs with a low risk of bias; 1– Meta-analyses, systematic reviews or RCTs, or RCTs with a high risk of bias; 2++ High quality systematic reviews of case-control or cohort studies or High quality case-control or cohort studies with a very low risk of confounding, bias, or chance and a high probability that the relationship is causal; 2+ Well conducted case-control or cohort studies with a low risk of confounding, bias, or chance and a moderate probability that the relationship is causal; 2– Case-control or cohort studies with a high risk of confounding, bias, or chance and a significant risk that the relationship is not causal*.

## Results and Discussion

### Search Results

PubMed searches yielded 1,163 “hits” for “circumcision women” and 2,994 hits for “circumcision female.” Google Scholar generated 1,000 hits (the maximum return for this search engine). EMBASE gave 3,201 and 740 hits for each respective search term, and the Cochrane database 1 and 2 hits, but neither generated additional citable articles. Publications pertaining to female “circumcision” more correctly termed “female genital cutting” or “female genital mutilation” (152) were excluded. Table 1 lists, by topic, relevant articles retrieved. This included 67 from PubMed, and one further article from Google Scholar. No further articles were found by EMBASE and Cochrane searches. Examination of bibliographies of the articles chosen yielded 6 further articles and 8 conference abstracts.

### Human Papillomavirus and Cervical Cancer

#### Observational Studies

Evidence that MC protects women against cervical cancer has accumulated over the years. In 1901, it was first noted that cervical cancer was uncommon in Jewish women (90), suggesting a cultural factor. A 1964 study in London of 288 predominantly Anglican patients found that women with cervical cancer were 4-times more likely to have ever been married to an uncircumcised man (85). No association with MC was, however, found in a 1973 New York study of 64 cases (95), nor for 198 husbands (12% circumcised) of women with cervical cancer in Central American countries (86).

Prior to HPV being implicated as the etiological agent in cervical cancer in 1983 (153), a role for smegma under the foreskin was suggested. Smegma is the whitish sebaceous secretion, with a cheese-like consistency, containing bacteria, other microorganisms, dead skin cells, mucous and other components. Injection into the vaginal canal of mice of horse smegma (91) or human smegma (bi-weekly for a year) (92, 93) was found to induce cervical cancer. This association was not seen for short-term exposure (94). A 1989 study of women found smegma in the male partner was associated with a 51% increase in cervical cancer risk (86).

A 1987 study found that women with cervical cancer were more likely to have partners with penile intra-epithelial neoplasia (PIN), the precursor to penile cancer (154). A 1991 Danish study found cervical cancer in 1 (14%) of 7 women with a circumcised partner compared with 39 (47%) of 83 women with an uncircumcised male partner (87). A study in Madras (now Chennai) of 5,000 cervical cancer cases between 1982 and 1990 noted a statistically significant lower incidence per 100,000 amongst Muslim women (24.5) compared with non-MC circumcising Hindu (78.3) and Christian (63.2) women (98). A case-control study of 137 women with persistent dysplasia who had only had sexual relations with one partner found sex with uncircumcised men or men circumcised after infancy was associated with a 4-fold higher risk of cervical cancer (96). This study controlled for factors such as age, age at first intercourse, and education. Research into various types of cancer in the Valley of Kashmir concluded that universal MC in the majority Muslim community was responsible for a cervical cancer rate that was lower than the rest of India (97). A 1994 study of women with the precursor of cervical cancer, squamous intra-epithelial lesion (SIL)—previously known as cervical intra-epithelial neoplasia (CIN)—found PIN in the male partner in 93% of cases (155). In Jordan, among attendees of obstetrics and gynecology clinics, circumcision of the male partner was associated with significantly lower SIL (103). No association with SIL or genital warts was found in a small Kenyan study in 2000 (36).

Compelling evidence from a well-designed multinational study in 2002 by the International Agency for Research on Cancer, published in the *New England Journal of Medicine*, implicated presence of the foreskin as a risk factor in cervical cancer (83). This study involved 1,913 couples in 5 locations in Europe, Asia and South America. Penile HPV was found in 20% of uncircumcised men, but only 5% of circumcised men (adjusted odds ratio [OR] = 0.37; 95% CI 0.16–0.85). After adjustment for age of male and female subjects, study location, age at first sexual intercourse, male education level, male's frequency of genital washing after intercourse, and male's lifetime number of sexual partners, the authors found that monogamous women whose male partner had a high sexual behavior risk index (6 or more sexual partners and first sexual intercourse prior to 17 years of age) had a 5.6 times lower risk of cervical cancer if their partner was circumcised (adjusted OR = 0.18; 95% CI 0.04–0.89). Monogamous women whose partner had an intermediate sexual behavior risk index (either ≥6 prior sexual partners *or* first sexual intercourse prior to age 17 years) had a 2.0 times lower risk of cervical cancer if their partner was circumcised (adjusted OR = 0.50; 95% CI 0.27–0.94). Penile HPV infection was associated with a 4-fold increase in risk of cervical HPV infection in the female partner. Cervical HPV infection was associated with a 77-fold increase in cervical cancer risk.

A Danish study that found 5-fold lower HPV prevalence in circumcised men concluded that, “*the female partners of circumcised men are less exposed to cervical cancer because these men are less likely to be infected with HPV*” (99). A 2006 study of UNAIDS data from 117 developing countries revealed a cervical cancer incidence of 35 per 100,000 women per year in 51 countries with a low (<20%) MC prevalence compared to 20 per 100,000 in 52 countries with a high (>80%) MC prevalence (*P* < 0.001) (84). Of all factors examined, absence of MC had the strongest association with cervical cancer incidence. A 2010 study found a complete absence of cervical cancer among Muslim women in rural India (100). The low cervical cancer prevalence in Israel, compared to global prevalence of 11.7% (5), was attributed in part to MC (102). Kuwait, predominantly Muslim, where males are circumcised prior to puberty, has an HPV prevalence of only 2.3%, one of the lowest in the world (101). A study in Myanmar of 200 women found cervical cancer was less common among women whose husband was circumcised (*P* = 0.025) (88). In Seoul, South Korea, MC in sexual partners was associated with a 53% reduced risk of invasive cervical cancer in women (OR = 0.47; 95% CI 0.24–0.90) (89). A Spanish study of 3,261 women found that those with two or more lifetime sexual partners had a 40% lower HPV risk if their male partners were circumcised (110). In a Nigerian study, all 8 women with uncircumcised male partners had positive HPV serology compared to 66% of women with circumcised male partners; the former were 13 times more likely to have abnormal cytology (105). A 2017 study in Ghana found elevated risk of high-risk HPV from having an uncircumcised partner amongst HIV-negative women [relative risk (RR) = 1.9; 95% CI 1.1–3.5; *P* = 0.03] and HIV-positive women (RR = 1.4; 95% CI 1.2–1.6; *P* < 0.0001) (111). A 2014 study in Kenya of women, 64% of whom were HIV-positive, found no association of MC and higher rates of low-grade and high-grade cervical abnormalities (104). A Nigerian study found 5% of 192 women with circumcised male partners had abnormal cytology compared with 63% of 8 women with uncircumcised partners, a 14-fold difference; the former also had 34% lower prevalence of HPV seropositivity (105).

A meta-analysis of 14 studies completed by September 2007 (5 in the US, 2 in Mexico, 2 in Australia, and one each in South Korea, Denmark, England, Kenya and the multinational study in Brazil, Colombia, Spain, Thailand, and The Philippines) found an OR of 0.75 (95% CI 0.49–1.14) for the association between MC and cervical cancer in monogamous women (82). The authors suggested the need to consider male partner risk rating as was done in the multinational study by Castellsague et al. discussed above (83).

#### Randomized Clinical Trials

RCTs are needed because association studies may suffer from confounding as a result of cultural differences other than MC practice—for example, extreme social sanctions against women having multiple or concurrent partners may be associated with slower HPV spread than that seen in communities without such sanctions.

Data from three RCTs provide definitive evidence for the ability of MC to protect men against high-risk HPV prevalence and incidence, and increase HPV clearance (156–159). The MC trial in Uganda simultaneously enrolled female partners. It involved 1,248 couples, with follow-up data obtained for 544 women in the intervention group (male partners circumcised) and 488 in the control (uncircumcised) group, and found high-risk HPV prevalence 24 months after MC was 27.8 vs. 38.7%, respectively [incident risk ratio (IRR) = 0.72; 95% CI 0.60–0.85; *P* = 0.001] (106). HPV incidence was 20.7 vs. 26.9 infections per 100 person-years (IRR = 0.77; 95% CI 0.63 to −0.93; *P* = 0.008). Overall, female partners of circumcised men had a 28% lower prevalence of high-risk HPV compared to those with uncircumcised partners (106).

In the Ugandan trial, genotype-specific HPV load in one partner was associated with risk of new detection of the same genotype in the other partner 1 year later (108). Women with circumcised partners had 58% lower incident HPV detection than women with uncircumcised partners. Female partners of circumcised men acquired fewer genotypes and also had increased clearance compared to the female partners of uncircumcised men. Decreased viral “*shedding*” among men was suggested as a reason for the reduced acquisition of HPV in female partners of the circumcised men (160). In uncircumcised men with a high viral load, clearance of high-risk HPV16 and HPV18 was lower, thus increasing risk of HPV transmission to female partners (112). In addition to the lower risk of oncogenic HPV infection, female partners of circumcised men in the Ugandan RCT had lower HPV viral loads that were contributed entirely by incident new high-risk HPV infections during the 24 months follow-up period (PRR = 0.66; 95% CI 0.50–0.87, *P* = 0.003) (113).

Circumcision of HIV-infected men did not affect transmission of high-risk or low-risk HPV to their female partners (109).

Low-risk HPV prevalence was 32% lower in female partners of circumcised men in the Ugandan trial (IRR = 0.68; 95% CI 0.53–0.89) (106).

#### Mode of Transmission

Genital HPV genotypes are highly infectious and can infect skin in the genital region. Skin-to-skin contact that does not necessarily extend to sexual intercourse could still result in infection (161). Redundant prepuce or phimosis, which together were an independent risk factor for HPV infection (OR = 3.4; 95% CI 2.5–4.6), was found in 78.6% of the male sexual partners of the 50.9% of women who had a HPV infection in a study in Nanjing, China (162).

Although uncircumcised men washed their genitals more frequently after intercourse in the multinational study of countries in Europe, Asia, and South America, circumcised men had better penile hygiene, as assessed by a physician (83). It was suggested that uncircumcised men are much more likely to be infected because during intercourse the more delicate inner (mucosal) lining of the foreskin becomes wholly exposed to vaginal secretions. Post-coitus, an infectious inoculum may become trapped within the preputial space and be transmitted from surface epithelial cells to basal layers where squamous changes to basaloid cells takes place. The vulnerability of mucosal surfaces to HPV infection also applies to the cervix, particularly the transition zone in women.

In summary, there is strong evidence showing that having circumcised male partners substantially reduces women's risk of HPV infection and, thus, lowers risk of cervical cancer, a HPV-dependent disease, and other genital cancers in which HPV makes a lesser etiological contribution.

### Herpes Simplex Virus Type 2

A 2003 study in Pittsburgh, Pennsylvania, of 1,207 women aged 18–30 years in which overall HSV-2 seroprevalence was 25%, found that a history of sexual intercourse with an uncircumcised man (ever) increased risk of HSV-2 infection 2-fold (OR from multivariate logistic regression analysis = 2.2; 95% CI 1.4–3.6) (116). HSV-2 prevalence was lower in 9 countries in which MC prevalence was >80% than it was in 10 countries in which MC was <20% (30.1 vs. 42.9%, respectively; *P* > 0.05) (84). A study in India found an HSV-2 prevalence of 1.7% among women with circumcised spouses compared with 9.2% among women with uncircumcised spouses (OR = 5.7; 95% CI 1.4–23.4; *P* = 0.01) (117). Similarly, a South African study of 4,766 women aged 15–49 years found that a circumcised male partner was associated with lower HSV-2 prevalence in both younger women (49 vs. 62%; *P* < 0.01) and older women (83 vs. 86%; *P* = 0.04) (119). Older women with circumcised male partners were less likely to have ever had an STI (7 vs. 11%; *P* < 0.01) (119). Among 8,953 women in Kenya, those with circumcised partners had an HSV-2 prevalence of 39.4% compared to 77.4% in women with uncircumcised partners (*P* < 0.001) (115). Risk of HSV-2 infection was 7.5 times higher among HIV-positive vs. HIV-negative women in that study. However, a study involving 14 sites in 7 sub-Saharan African countries found that MC provided negligible protection against HSV-2 infection among female partners (PRR = 0.94; *P* = 0.49) (118). A non-significant 15% risk reduction was found in the female partners of 368 circumcised men in a RCT in Uganda (114). In a Kenyan study, there was no difference in HSV-2-related genital ulcers between 14 women with circumcised and 14 women with uncircumcised partners (120).

In summary, there is evidence from large observational studies, but not from trial data and some other studies, to suggest that MC may reduce HSV-2 infection and prevalence in women.

### Genital Ulcer Disease

Women with circumcised male partners in the Ugandan MC RCT had a 22% lower risk of genital ulcer disease (adjusted PRR = 0.78; 95% CI 0.63–0.97) (123). However, subsequent analyses found no difference (122). Secondary data from another RCT, in which the etiological agent was determined in 67% of genital ulcers in women, demonstrated HSV-2 as the primary pathogen in 96% of those women (121). Most women with HSV-2 were already infected at trial commencement, and HSV-2 detected in their swabs represented reactivation of existing infections (121). A small Kenyan study in 2000 found no association between partner MC and HSV-2 in women (36).

### Chlamydia trachomatis

A multinational study of 305 couples in 5 diverse countries globally found a 5.6-fold increased risk of *C. trachomatis* antibodies among women with an uncircumcised male partner having had 6 or more sexual partners compared to women with a circumcised partner with a similar sexual history (124). Assessing lifetime exposure by measuring antibodies, this study found that the frequency of antibodies to *C. pneumoniae*, a species of chlamydia that is not sexually-transmitted, did not differ between women with circumcised vs. uncircumcised partners, supporting the protective role of MC in reducing C. *trachomatis* infection risk in women (124).

A study in India found a *C. trachomatis* prevalence of 9% among women with circumcised male partners vs. 21% in women with uncircumcised male partners (127). A multivariate analysis of serology data in a Pittsburgh and North Carolina study found a 2.6-fold higher risk of *C. trachomatis* among women who had had sexual intercourse with an uncircumcised male in the previous 3 months (95% CI 1.21–5.82) (126).

Cervicovaginal secretions infected with *C. trachomatis* that become trapped under the foreskin in uncircumcised men could increase the risk of penile urethral infection and onward *C. trachomatis* transmission to women during sexual intercourse (124). However, a meta-analysis of observational study data in men suggested that MC does not protect against Chlamydia and other urethral STIs (163). RCT data in men have been conflicting, with a significant 44% reduction found in the South African MC RCT (164), but no significant reduction found in the Kenyan MC RCT (165).

Other studies in women have failed to find an association between *C. trachomatis* infection and partner MC status. These include a small Kenyan study in 2000 (36), a 2016 study in Kenya, Uganda, and South Africa (128), and a prospective study of 5,925 women in Uganda, Zimbabwe and Thailand in 2008 (125).

In summary, there is evidence for and against MC being associated with reduced *C. trachomatis* infection risk in women. Given the absence of consistent RCT data for *C. trachomatis* in women, further trials are warranted.

### Neisseria gonorrhoea

No association between MC status and gonorrhea in female partners was found in a small study in 2000 from Kenya (36), nor in a study of 5,925 women from Uganda, Zimbabwe, and Thailand (125), or in a study of women in Kenya, Uganda, and South Africa in 2016 (128). However, in a 2014 study in India involving 61 women, gonorrhea was absent in women with circumcised male partners, but was present in 7.1% of women with uncircumcised partners (127).

In summary, there is little evidence to suggest that partner MC status reduces gonorrhea risk in women. This is to be expected because MC status has no effect on sexually transmitted urethritis in men (163).

### Trichomonas vaginalis

Results from the Ugandan RCT documented a 48% reduction in *Trichomonas vaginalis* prevalence (adjusted PRR = 0.52; 95% CI 0.05–0.98) in 825 wives of circumcised men compared to 783 wives of uncircumcised men (123). It was suggested that the infection pathway may involve the moist subpreputial space, potentially enhancing *T. vaginalis* survival and transmission (123). Amongst 981 couples, from 14 sites in 7 African countries, in whom at least one partner was infected, women with circumcised male partners had an 18% (*P* = 0.004) lower risk of *T. vaginalis* infection in multivariate analysis. A smaller Ugandan study of 138 and 112 female partners of circumcised vs. uncircumcised men found a trend to lower *T. vaginalis* prevalence in women of 7 vs. 15%, respectively (*P* = 0.056) (129). No association was, however, found in a study in 2000 of 520 women in Kenya (36), nor in a multinational study in 2008 (125).

In summary, based on RCT data in particular, it is possible that MC may be associated with reduced risk of trichomoniasis in women, but further research is warranted.

### Syphilis (*Treponema pallidum pallidum*)

A small Kenyan study in 2000 found no association between MC status and syphilis infection in women (36). Amongst 663 pregnant women in Tanzania, women's syphilis seroprevalence was lower in those with circumcised than uncircumcised male partners [1.3 vs. 4.7%; univariate and multivariate OR = 3.8 (*P* = 0.001 and 0.01, respectively)] (131). A study in Kenya, Uganda and South Africa of 1,561 women with circumcised partners and 2,863 with uncircumcised partners found a reduced risk of syphilis [hazard ratio (HR) = 0.51; 95% CI 0.26–1.00, *P* = 0.058], when partners were circumcised, although there were important differences in the number of reported sex acts in the previous 7 days and the use of condoms at last sex act that may have affected the findings (128). A South African study of women aged 15–49 years found male partner MC status was negatively associated with syphilis serology (1.5 vs. 3.4%; *P* = 0.04 for circumcised vs. uncircumcised partners, respectively) (119). A large prospective cohort study of 2,946 HIV-negative couples found syphilis was 75% lower among female partners of circumcised men (130). In a small Indian study, syphilis was absent in women with circumcised male partners vs. 3.5% for women with uncircumcised male partners (127).

Together, these findings provide evidence to suggest that MC may help protect women against syphilis. Additional studies are needed to establish the precise magnitude of this protective effect.

### Chancroid (*Hemophylus ducreyi*)

We found no studies of MC and chancroid in women. It has, however, been noted that in southern and eastern parts of Africa where prevalence of MC is low and HIV is high, chancroid is endemic, that chancroid is closely associated with sex work, and that chancroid is a strong risk factor for HIV infection (166). In recent years chancroid has decreased in prevalence in many parts of the world (167).

### Human Immunodeficiency Virus

Findings concerning potential HIV risk reduction in women by sexual partner MC status have varied. In Rwanda a 1994 study found a statistically significant higher HIV seroprevalence in pregnant women with circumcised vs. uncircumcised partners (24.4 vs. 8.4%), being similar (each 2.2-fold) for monogamous and high-risk women after multivariable regression analysis (132). In contrast, no association was reported in a Rwandan study in 1991 (143), Higher socioeconomic status and multiple sexual partners were also risk factors when controlling for other covariates. A 1994 study of 4,404 women in Kenya found a 2.9-fold lower HIV prevalence among women with circumcised vs. uncircumcised partners (4.2 vs. 11.5%) (133). Most women in that study reported only one partner in the previous year. HIV risk related to partner MC status occurred in almost all strata of potential confounding factors in the study. A 1998 study in Dar es Salaam, Tanzania, reported a 4-fold higher relative risk of HIV for married women with one sex partner if their husband was uncircumcised (134). A 2000 study in Kenya found HIV prevalence was 2-fold higher in women with uncircumcised partners (36). Although a 31% lower HIV prevalence was found for Zimbabwe women with circumcised partners, adjustment for potential confounding factors rendered the relationship non-significant (135). A 2010 study of 1,096 serodiscordant couples from 7 sites in eastern Africa followed for 18 months found that female partners of HIV-positive circumcised males had a 38% (*P* = 0.10) lower HIV prevalence (137).

A RCT in Rakai, Uganda, of uninfected female partners of men who were already infected with HIV at the time of their circumcision, documented 17 infections in women whose partner became circumcised at baseline and 8 in women whose partner's circumcision was delayed for 24 months (18 vs. 12%; adjusted hazard ratio 1.49; *P* = 0.37) (129). Since MC of HIV-infected men did not reduce HIV transmission to female partners over 24 months, the trial was stopped for “*futility*” at interim analysis. Thus, “*longer term effects could not be assessed*.” Although no statistically significant differences were found overall, it was notable that at the first follow-up visit at 6 months, 28% of female partners of circumcised men who resumed sex before wound healing had acquired HIV compared to 9.5% of those whose male partners had delayed sex until healing was complete (*P* = 0.038) (129). Resumption of sex prior to the recommended 6-week healing period was an important factor in the interpretation of these findings (129, 168). It was later calculated that 10,000 serodiscordant couples would need to be enrolled to detect a significant effect, a task deemed “*logistically unfeasible*” (169).

Amongst 657 pregnant women in Zimbabwe and Tanzania, HIV was not significantly different in women whose partner was circumcised vs. uncircumcised (7.1 vs. 11.5%, respectively) (144). In a study of 13 sub-Saharan African countries, regional MC prevalence of MC was associated with significantly lower HIV prevalence among women (adjusted OR = 0.27 and 0.10 for medium and high MC prevalence, respectively, vs. low MC prevalence; each *P* < 0.001) (136). In southern, but not northern, rural Malawi where HIV prevalence is highest, women with circumcised partners had half the HIV prevalence of other women (*P* < 0.05 in logistic regression models) (138). A study of 2,223 women from 14 sites in eastern and southern Africa followed for ≤ 24 months, found that male partner circumcision status was significantly associated with a 47% lower HIV prevalence in the women (139). Observational studies involving men circumcised before puberty suggest a stronger protective effect for women than do the RCTs data from men circumcised as adults (147). Among 5,561 women ever having had sexual intercourse, HIV prevalence was lower for the 30% who had only ever had circumcised partners (22.4 vs. 36.6%; adjusted PRR = 0.85; *P* = 0.004) (141).

One study in Tanzania found that in areas where most males are circumcised, the HIV burden could be higher among women, leading to a recommendation that along with VMMC, HIV prevention efforts engaging women are needed (79). Another Tanzanian study found a non-significantly 18% lower HIV prevalence among pregnant women with circumcised partners (131).

A meta-analysis of data from one RCT and 6 longitudinal studies to August 2009 found that MC was associated with a non-significant 20% HIV reduction in women (summary RR = 0.80; 95% CI 0.54–1.19) (169). A 2015 meta-analysis limited to RCTs and cohort studies found a 32% non-significantly lower HIV risk in women with circumcised male partners (pooled adjusted RR = 0.68; 95% CI 0.40–1.15; *P* = 0.15) (107).

In the setting of a VMMC rollout in Orange Farm (the site of the previous South African MC RCT), among 4,538 women aged 15–49 a significant 16.9% reduction (adjusted IRR = 0.83; 95% CI 0.011–0.69) was found in those who only had circumcised male partners (140). Another South African study, however, found that, after adjustment, MC was not associated with reduced HIV risk in women (146). A South African cohort study of 1,356 HIV-negative pregnant women found a 78% lower adjusted HIV prevalence that was not statistically significant among women with circumcised vs. uncircumcised partners (142). A further South African study involving 4,766 women, found partner MC was associated with significantly lower HIV prevalence in women (24 vs. 35%; *P* < 0.01) (119).

In comparable high-income countries, the prevalence of heterosexually acquired HIV prevalence in countries with low MC prevalence (the Netherlands and France) was 10 times higher in women (and 6 times higher in men) than in Israel, a country with a very high MC prevalence (145).

Intuitively, it has been thought that if a man is HIV-positive, whether he is circumcised or not, should make little difference to HIV infection risk for his sexual partners (135). An exception, however, was found in the case of women from high-risk settings (HR = 0.16). A study in Uganda in 2000 found viral load of <10,000 and 10,000–49,999 copies/ml in the male partner was associated with a HIV incidence/100 person-years of 6.9 (95% CI 2.8–11.0) and 12.6 (95% CI 6.8–18.4), respectively, in women with uncircumcised male partners (61). No infection was evident in women with circumcised male partners. But if the male had a HIV viral load ≥50,000 copies/ml, HIV incidence per 100 person-years did not differ: 25.0 (95% CI 0.50–49.5) vs. 25.6 (95% CI 15.4–35.8) in women with circumcised vs. uncircumcised partners (61).

### Mechanism of HIV Infection in Women

Several characteristics of the female genital tract increase the likelihood of HIV acquisition following exposure, establishment of infection, and systemic spread of the virus, causing local changes that favor infection by other STIs (170). This bidirectional synergistic relationship between HIV and infection with other STIs increases local replication of HIV (171). Not only has HIV been isolated from surface ulcers in the genital tract of HIV-positive women, HIV viral shedding is increased in women with genital ulcers (172) and genital ulcers increase HIV risk in women 8.5-fold (173). However, HSV-2 suppressive therapy for women in a RCT did not decrease their risk of HIV acquisition (174).

Regardless of whether MC has a direct effect or not on HIV transmission to female partners, there will be indirect benefits to MC scale-up for women as the pool of men living with HIV declines in the long-term, reducing HIV transmission to women (175, 176). UNAIDS has estimated that, in Africa, for every 5% increase in MC, HIV infection rates would decrease by 2% in women (177).

In summary, although MC does not appear to directly reduce HIV infection in women, because it reduces HIV acquisition in men, it reduces the population-level prevalence in men (see section Introduction for references), and this reduces the risk of infection risk for women.

### Bacterial Vaginosis

A longitudinal US study in Pittsburg of 773 women without BV at enrolment found that, over 1 year, those with uncircumcised partners were twice as likely to develop BV (148). An RCT in Uganda found that BV in general was 40% lower (adjusted PRR = 0.60; 95% CI 0.38–0.94), and severe BV was 69% lower (adjusted PRR = 0.31; 95% CI 0.18–0.54), among the wives of circumcised men (123). There was no difference in BV rates in female partners of HIV-infected men in this setting (129). No association was found in a small study in 2000 from Kenya (36). In an Indian study, BV was found in 4.5% of women with circumcised partners compared with 7.1% of women with uncircumcised partners (127). Two small US studies with low power found no association between women's BV risk and their male partner's circumcision status (149, 150).

The male foreskin could facilitate survival of BV-associated organisms, such as gram-negative anaerobic bacteria, resulting in more efficient and prolonged transmission of such bacteria to sexual partners (39). The much higher prevalence of gram-negative anaerobic bacteria under the foreskin of men prior to MC was evident from the microbiome of men in a large RCT (147). MC, by reducing penile proinflammatory anaerobic bacteria, decreases BV risk in the female partners (147).

Genital ulcers from women with an uncircumcised male partner contained a higher prevalence of presumed bacterial agents of BV (120). Of 14 bacterial taxa, only *Gardnerella* taxa differed significantly (71% reduction) by male partner MC status (120).

A meta-analysis found a positive association between BV and periodontal disease (178).

Receptive oral sex with an uncircumcised partner was associated with 1.3-times higher risk of periodontal disease than with a circumcised partner (178).

In summary, most evidence, including high quality RCT data, points to MC being associated with a reduced risk of BV in women.

### Other STIs and Conditions

Candida was 40% lower in 520 women in Kenya whose male partners were circumcised (OR = 0.6; 95% CI 0.4–1.0) (36). No reduction in female partners was found for *Mycoplasma genitalium* (122), dysuria (123, 129), and vaginal discharge (122, 123, 129).

### MC in the Context of Other Means of STI Reduction

#### Condoms

Condoms offer protection against various STIs, with effectiveness differing between different STI types and consistency of use (179). Condoms provide 80% (180) to 71–77% (181) protection against HIV infection if used consistently and correctly (180, 182). A study in Mysore, India, found that among the 40 women whose partner used a condom at last sexual intercourse, HIV prevalence was non-significantly higher than in 2,225 women whose partner did not use a condom (adjusted OR = 10.5; 95% CI 2.05–53.8; *P* < 0.01 after multivariate analysis) (183). Among men in this study, ever having used a condom was associated with higher HIV prevalence (adjusted OR = 2.7; 95% CI 1.0–7.5; *P* = 0.05). Only 12% of men reported ever having used a condom. An explanation offered for these findings was that people with high-risk behavior first become infected by HIV then, after learning about their HIV infection and becoming aware of their high-risk behavior, begin to use condoms. The same reasoning was used to explain why condom use was not associated with a reduced odds of HIV infection in a study of 13 African countries (136). A Cochrane systematic review and meta-analysis of RCTs of condom use (2 in the US, one in England and 4 in Africa) found, “*little clinical evidence of effectiveness”* and no “*favorable results”* for HIV prevention (184). Diaphragms—used commonly by women as a contraceptive—provide no protection against HIV infection (185).

In the multinational study by Castellsague et al., the effect of MC on cervical cancer reduction differed little between condom users (OR = 0.83; 95% CI 0.37–1.87) and non-users (OR = 0.67; 95% CI 0.44–1.02) (83). In this and other studies, condoms offered only slight protection against HPV infection (83, 106, 110). A study of university undergraduates in Seattle, however, found 70% lower HPV incidence in women whose partners always used condoms compared with those whose partners used condoms <5% of the time (186). Squamous intraepithelial lesions were absent in women whose partner always used condoms, compared with 14 per 100 person-years in non-users. In women who had been treated for squamous cervical lesions, consistent condom use reduced high-risk HPV infection by 82% (95% CI 0.62–0.91) (187). A 2014 systematic review found 4 studies in which condoms provided statistically significant protection against HPV infection and 4 in which protection did not reach statistical significance (188).

Condom use did not protect women against HSV-2 in a large Kenyan study (115). A Cochrane analysis found that condoms were 42% effective in prevention of syphilis infection (184). In a US study, using condoms with occasional partners provided protection against BV (RR = 0.80; 95% CI 0.67–0.98; *P* = 0.003) (149).

In the US, 16% of men and non-primary partners of 24% of women never used condoms during heterosexual sex (189). A US survey of women attending STI clinics in Baltimore found that consistent condom use was 25%, with 48% saying there had been no condom use in the previous 14 days (190). Testing for male DNA in the vagina, however, showed that DNA was present in all women, albeit being higher in the group who stated that condoms were not used (190). A review in *the Lancet* in 2000 reported condom use was 55% (191). Amongst younger people, an Australian study found only 25% always used condoms, with 25% never having used them (192). A survey in Mexico found young men reported condom use of 51%, whereas young women reported it as 23% (193). Consistent condom use was only 30% in this study (193). In 13,293 Mexican public school students, in whom average age of sexual debut was 14 years, 37% had a high and 46% had an intermediate HIV/AIDS knowledge (194). Males with high knowledge were more likely to use condoms (OR = 1.4), whereas females in this category were less likely to ensure the male partner used condoms (OR = 0.7) (194). In a RCT in Uganda involving the female partners of HIV-positive men, it was found that, despite undergoing counseling to use condoms, 61% of the sexually active participants did not use them at all, 20% used them inconsistently, and only 20% used them always (129). At each follow-up visit in this 2-year RCT, the majority did not use condoms.

These studies show that the evidence about the effectiveness of condoms for STI prevention is mixed. Condoms are an important part of the STI prevention toolbox, yet to be effective, they require correct and consistent use over a lifetime. In contrast, MC is a one-off procedure that does not require action each time a man has sexual intercourse. When both MC and condoms are in place protection is higher (195). The issue is that it is exceedingly difficult to ensure consistent and correct condom use in the most vulnerable populations.

#### Pre-exposure Prophylaxis (PrEP) for HIV Prevention

PrEP with anti-retroviral medication has proven to be very effective in study settings, and is being rolled out in several countries. Cost and health systems implications of providing PrEP for possibly decades presents challenges. In 2017 there were 36.9 million people globally living with HIV, 21.7 million accessing anti-retroviral therapy and 1.8 million newly infected with HIV (196). Attempts, moreover, to develop a microbicide for use by women have been unsuccessful (197–206).

#### Vaccination

The only vaccine against an STI is for HPV. Starting in 2007, prophylactic vaccines against HPV 16 and 18 (present in ~70% of cervical cancers) and, in the case of the quadrivalent vaccine, low-risk HPV 6 and 11, have become available for administration to girls early in high school or at an equivalent age for girls who do not attend school (207). Not only has this reduced vaccine-type HPV prevalence in females, but the female vaccination program has had a flow-on herd immunity effect reducing HPV infection in males (208). In 2013 the program was extended to boys of the same age and is expected to further reduce HPV 16 and 18 infections in both sexes. A systematic review found that in Australia, one of the earliest countries to vaccinate girls (with the quadrivalent HPV vaccine) there has been a 76–80% (not 100%) 10-year reduction in HPV types 16 and 18 in females aged 18–24 years, with lesser reductions in the US and European countries (207).

In the US in 2007, prior to vaccine adoption, HPV16 was the 6th most common high-risk HPV type and HPV18 was 10th (209). Baseline estimates of US population prevalence of 37 HPV genotypes prior to HPV vaccination was 27% overall, reaching a maximum of 52% at age 20 years (210). Prevalence of HPV16 was 9.6, 6.5, and 1.8% in age groups ≤ 20, 21–29, and ≥30 years (210). HPV16 and/or HPV18 prevalence was 12, 8.3, and 2.4% in each respective age group (210). These two HPV types were present in 54.5% of high-grade squamous intraepithelial lesions. Modeling suggested that 80% vaccine coverage in girls should result in a 55% overall reduction in high-risk HPV prevalence (211). Modeling of the current girls-only vaccination coverage of 60% in the Netherlands was estimated to reduce cervical cancers and deaths by 35% more when compared to primary screening in the absence of vaccination (212).

Genital HPV infection is occurring at a younger age in many countries. In the UK prior to the HPV vaccine era, 5% of girls under 14 had HPV antibodies, indicating current or prior infection (213). By age 16 the proportion infected was 12%, by 18 it was 20%, and by age 24 it was 45%, with subsequent decreases thereafter (213). Oncogenic HPV16 was the most common type. In the US, 7% of girls aged 12–19 years had HPV16 antibodies, rising to 25% for those aged 20–29 years (214). Rates of other STIs are also rising in teenagers in developed countries.

Elimination of HPV 16 and 18 from the population by vaccination might take decades. At the population level, it has been suggested that other oncogenic HPV types not included in the vaccines might take over and replace these two types of HPV (210, 215, 216). There is now evidence that this is occurring. Eight years after introduction of the HPV vaccination program for girls in Australia, although prevalence of HPV16 together with HPV18 in heterosexual men decreased from 13 to 3% (*P* < 0.0001), there was no decrease in HPV genotypes overall, and prevalence of non-vaccine-targeted genotypes increased significantly from 16 to 22% (*P* < 0.0001) (208). A US study found non-vaccine-types of HPV increased from 61 to 76% (*P* < 0.0001) in vaccinated young women 4 years after vaccination (217). Vaccination was, moreover, accompanied by a reduction in rate of participation in cervical screening programs in Australia by 20–24 year-old (37.6 vs. 47.7%) and 25–29 year-old (45.2 vs. 58.7%) women (218).

There is uncertainty about the long-term durability of the benefits of vaccination. Introduction of a nonavalent HPV vaccine, which will protect against additional high-risk types 31, 33, 45, 52, and 58 (meaning ~90% coverage), will increase the percentage of preventable HPV-associated cervical cancers from 66.2 to 80.9%, assuming 100% coverage and efficacy (219). Concerns about breadth of protection, adherence and long-term immunity will remain. Vaccination has a much smaller effect against vulval epithelial neoplasia (220), oncogenic HPV types being present in only half of cases. Since HPV vaccines are not fully protective, cervical screening will need to continue (221).

While some countries have achieved success in their HPV vaccination programs, for example, Australia, which is on track to “eliminate” cervical cancer within two decades as a result of its national HPV vaccination program (222), participation in vaccination programs has been impeded by conservative and religious groups who falsely claim that vaccination of their daughters early in high school will lead to an increase in promiscuity. Studies in Canada and the US have found that amongst young adolescent girls, the HPV vaccine has no effect on sexual risk behavior (223), and has no significant impact on sexual-activity related outcomes (223). Nevertheless, there is low parental support in the US for routine HPV vaccination of young adolescents (224). Another impediment has been scaremongering by vigorous anti-immunization lobby groups in the news media and on the Internet. Most of the adverse events touted are not related to the vaccine, and would be seen in any large-scale vaccination program by pure coincidence. For example, Gullain-Barre syndrome has been suggested as an adverse effect of the HPV vaccine, although post-HPV vaccine, the prevalence of this condition (0.06%) is low, and no greater than for other vaccines (225).

While prophylactic HPV vaccines will reduce cervical cancer incidence and deaths, they do not cover the full spectrum of oncogenic HPV types. In contrast, MC partially protects against *all* oncogenic HPV types (106).

In summary, the data support a combination of personal and public health measures that should be advocated for STI and genital cancer prevention—here, MC, vaccination, and condom use should be seen as working synergistically. MC and vaccination should preferably be delivered before sexual debut (226) and condoms encouraged once sexual activity has begun.

### Acceptability of MC Among Women

A review of 13 studies from 9 sub-Saharan African countries found 69% of women (range 47–79%) favored MC for their partners (227). Furthermore, 81% (70–90%) of women were willing to get their sons circumcised. A study in South Africa found that women had a strong influence on the decision, often scheduling the appointment for MC for their boyfriend or husband (228). The authors of the South African RCT in 2005 suggested an important role of awareness by women of the protective effect of MC in encouraging males to become circumcised (64). Particularly in regions of high HIV prevalence, the majority of uncircumcised men want the procedure performed, and generally an even higher proportion of women in those regions would prefer to have a circumcised partner (229). Interest in MC in the first VMMC roll-out programs in Africa was in part driven by women's preference for circumcised partners (168). In Botswana, 92% of mothers of newborn boys wanted their sons to be circumcised if the procedure was available in a clinical setting, with 85% saying the father must participate in the decision (230).

In the setting of the RCT in Orange farm, South Africa, preference by women for a circumcised partner increased from 64.4% in 2007 (*n* = 1,258) to 73.7% in 2012 (*n* = 2,583) (231). Preference for circumcision of male children increased from 80.4 to 95.8% during this period. Among 2,581 women having had sexual intercourse with circumcised and uncircumcised men, 55.8% agreed that it was easier for a circumcised man to use a condom, 20.5% disagreed and 23.1% did not know. It has been suggested that MC, “*could be incorporated rapidly into the national plans of countries where most males are not circumcised”…*. just as in South Korea where MC has risen from virtually zero 50 years ago to 85% today (232).

Mathematical modeling has predicted that with 80% MC uptake, a 45–67% reduction in HIV prevalence would be achieved in both men and women within a decade in African countries with high HIV prevalence (233). With a 50% uptake, HIV would be reduced 25–41% (233). Further modeling has predicted that for a 60% efficacy, 19 circumcisions would prevent one HIV infection in both sexes at a cost of $1,269 per infection averted (234).

In sub-Saharan African countries, adolescent females played a role in convincing young males to participate in VMMC programs, were supportive of their decisions to participate, and were supportive during the wound-healing process (235, 236). They used their romantic relationships or the potential for sex as leveraging points (235, 236). They believed VMMC to be beneficial for the sexual health of both partners and viewed males with a circumcised penis as more attractive (235). Improved penile hygiene and increased sexual pleasure were other reasons (236). Women did not exhibit risk compensatory behaviors (such as having unprotected sex or more partners) after their male partner was circumcised (237).

### Limitations

Cultural differences in MC and female sexual exposure to STIs may limit the interpretation of observational study data. Such limitations can be overcome by RCTs. While RCT data has established that male partner circumcision reduces women's risk of HPV, *T. vaginalis*, bacterial vaginosis and possibly genital ulcer disease, there are no RCT data for HSV-2, *C. trachomatis, Neisseria gonorrhoea, Treponema pallidum, M. genitalium*, candida and dysuria in women. RCT data for cervical cancer are also lacking, although such a trial would require many years and would likely be deemed unethical given the RCT data showing HPV risk reduction in the female partners of circumcised men.

### Future Research Directions

In the US, HPV prevalence in oropharyngeal cancers in adults has increased significantly from 16.3% during 1984–1989 to 71.7% during 2000–2004 (238). It is predicted that 49,750 newly diagnosed oropharyngeal cancers will be recorded in the US in 2018, with a 5-year survival of 57% (239). HPV can be transmitted to the mouth during oral sex (240). A large multinational study found that HPV was involved in ~20% of oropharyngeal cancers worldwide, with higher prevalence in countries with low MC rates (241). Since uncircumcised men are more likely to be infected with HPV, oral sex with uncircumcised men would likely increase infection and oropharyngeal cancer risk. No research studies have, however, examined the extent to which MC may be associated with increased oropharyngeal cancer risk. Such studies are needed.

HPV infection in men is associated with increased risk of anal cancer in women (242), either directly through anal sexual contact or indirectly through spreading of HPV from a cervicovaginal locus or other anogenital sites.

High-risk HPV genotypes have been found in breast tumors (243, 244) and can match those in the cervix of women with cervical cancer (245, 246). This suggested possible HPV transmission during sexual activities as a cause of some breast tumors (247). In support of this, women with HPV-positive breast cancers were significantly younger than those with HPV-negative breast cancers, in line with the higher risk of sexually acquired HPV infection among younger women (248). HPV-infected squamous epithelial cells (koilocytes) have been found in breast skin as well as in lobules from ductal carcinoma *in situ* and invasive ductal carcinoma (249, 250). Furthermore, HPV has been found in the bloodstream of cervical cancer patients (251). An Australian study of blood donors documented HPV attached to peripheral blood mononuclear cells, leading to the suggestion that blood could represent a viral reservoir and a potential new route of transmission (252). A viral etiology for breast cancer has also been invoked for mouse mammary tumor virus (MMTV) and Epstein-Barr virus (EBV) (244).

A meta-analysis found a significant association of HPV infection with lung cancer in men and women—HPV16 and HPV18 together showed a 9.8-fold higher risk for lung squamous cell carcinoma (253). Lack of data on tobacco consumption in the 9 studies analyzed precluded adjustment for that risk factor.

More research is needed to establish the role of HPV in the etiology of these other cancers and whether MC could reduce risk of some in women.

## Conclusions

This systematic review of the scientific evidence to date identifies MC as a potentially powerful tool to reduce the global burden of STIs on women. This review documents a range of effectiveness for MC against different STIs in women. Based on the highest quality evidence from RCTs, it can be concluded that MC reduces risk of oncogenic HPV genotypes, cervical cancer, *T. vaginalis*, bacterial vaginosis and possibly genital ulcer disease in women (Table 2). For other STIs in women the evidence regarding MC is variable or negative.

**Table 2:** RCT findings for STIs that MC protects women against.

**STI**	**Circumcised partner vs. uncircumcised partner**	**References**
High-risk HPV	Incident RR: 0.72 (95% CI 0.60–0.85; *P* = 0.001)	(106)
Genital ulcer disease	Adjusted prevalence RR: 0.78 (95% CI 0.63–0.97)	(123)
*Trichomonas vaginalis*	Adjusted prevalence RR: 0.52 (95% CI 0.05–0.98)	(123)
Bacterial vaginosis	Adjusted prevalence RR: 0.60 (95% CI 0.38–0.94)	(123)

Since MC is a highly effective, affordable and feasible as an STI prevention tool in men, reduced population prevalence of a number of STIs in men will translate into lower risk of STI exposure in women. MC's benefits to women were recognized by the US CDC in its 2018 policy arising from a detailed review (58). Scaling up MC more widely beyond HIV prevention programs is warranted, accompanied by increased investments in efforts to raise public awareness of its protective power. A combination of public health measures is necessary for effective STI prevention.

Lack of MC clearly represents an important risk factor in the worldwide epidemic of cervical cancer. Countries with declining MC prevalence, whether as a result of changes in public health policies on MC or increases in immigrants from non-circumcising cultures, are likely to experience an increase in cervical cancer incidence among women. Such increases may be partially ameliorated by HPV vaccination programs.

Males can be circumcised at any age. Maximum lifetime protection, minimal risk of adverse events, cost considerations, speed, faster healing, optimal cosmetic outcome and convenience applies to MC performed in early infancy (254). In its 2012 policy recommendations, the American Academy of Pediatrics suggested that parents should be presented with the scientific evidence in an unbiassed manner, and should be free to either consent or decline to having their son circumcised (255).

Women can have considerable power in regard to the MC decision for men and boys. They can influence the choice of MC early in infancy or later in life for their sons (254), brothers, other male family members and friends (256). They can, moreover, choose to have a circumcised sexual partner or encourage an uncircumcised partner to undergo the procedure (256). Doing so will help reduce STIs and a lifetime of assorted medical problems in males from infancy to old age, and help reduce the risk of certain STIs and of cervical cancer in women.

MC should be considered as a key component of a package of measures to reduce STI risk, with each component working synergistically. For example, MC and HPV vaccination delivered before sexual debut will have maximum effect (226). Public health recommendations that include HPV vaccination and MC, as well as other proven measures known to reduce STI risk, such as condoms, sexual partner reduction and PreP, especially in high-risk HIV settings, are well-anchored in the scientific evidence.

## Author Contributions

BM performed the literature review, evaluated the data, and drafted the manuscript. CH, JB, EL, AM, JDK, and JNK checked the data and made substantial contributions to successive drafts.

### Conflict of Interest Statement

BM held US Patents 5,783,412 and 6,218,104, European Patent 88902077.2-2107 (British, 0357611; German, P 3853678.1; Swiss, 3853678.1; Swedish, 0357611), Japanese patent 3096704 and Australian Patent 611135, with Priority date 26 Feb. 1987, for use of polymerase chain reaction technology for HPV detection in cervical screening. These expired in 2007. He is a member of the Circumcision Academy of Australia, a not-for-profit, government registered, medical association that provides evidence-based information on its website about MC and a list of doctors who perform MC in Australia and New Zealand. The authors have no religious or other affiliations that might influence the topic of MC. The remaining authors declare that the research was conducted in the absence of any commercial or financial relationships that could be construed as a potential conflict of interest.
